# Patient satisfaction with antiretroviral therapy services and associated factors at Gondar town health centers, Northwest Ethiopia: an institution-based cross-sectional study

**DOI:** 10.1186/s12913-020-4934-z

**Published:** 2020-02-06

**Authors:** Getaneh Adissu, Gashaw Andarge Biks, Koku Sisay Tamirat

**Affiliations:** 1 Federal Democratic Republic of Ethiopia, Pharmaceuticals Fund and Supply Agency, Addis Ababa, Ethiopia; 2 Department of Health System and Policy, College of Medicine and Health Science, Institute of Public Health, University of Gondar, Gondar, Ethiopia; 3 Department of Epidemiology and Biostatistics, College of Medicine and Health Science, Institute of Public Health Science, University of Gondar, Gondar, Ethiopia

**Keywords:** Patient satisfaction, Antiretroviral therapy services, HIV/AIDS, Health centers

## Abstract

**Background:**

The Human Immunodeficiency Virus (HIV) with which over 37 million peoples are living is the leading cause of morbidity and mortality worldwide. The rapid expansion of antiretroviral treatment has dramatically reduced HIV related deaths and transmissions. Patient satisfaction could be an indispensable parameter used to measure patients’ desired fulfillment by the services. Hence, this study aimed to determine the level of patient satisfaction with antiretroviral therapy services and determinants at Gondar town health centers.

**Methods:**

An institution-based cross-sectional study was conducted from November 1 to 30, 2018. The systematic random sampling technique was used to select 663 HIV/AIDS patients on antiretroviral therapy follow-ups. Data were collected using a pretested interviewer-administered questionnaire and patient medical document reviews. Summary statistics such as means, medians and proportions were calculated and presented in the form of tables, graphs, and texts. Bivariate and multivariable logistic regression analysis was fitted and adjusted odds ratio (AOR) with a 95% confidence interval (CI) was computed to assess the strength of association. Variables with *p*-value 0.05 at multivariable logistic regression considered significant determinants of patient satisfaction.

**Results:**

The overall patient satisfaction with antiretroviral therapy services was 75.4% (95%CI, 71.9 to 79%). Patients’ age 38–47 years (AOR = 5.90, 95%CI: 3.38,10.31) and ≥ 48 years (AOR = 2.66, 95%CI:1.38,5.12), absence of signs and directions to ART clinic (AOR = 0.53,95%CI:0.35,0.82), Azezo health center (AOR = 2.68,95%CI:1.47,4.66) and Teda health center (AOR = 4.44,95%CI:1.73,11.30), and travel that took more than 1 h (AOR = 0.56;95% CI:0.32,0.97) were determinants of patient satisfaction with the services.

**Conclusion:**

The overall patient satisfaction with antiretroviral therapy service was lower than the national target of 85% with the marked difference among health centers. Older age, absence of signs and directions to ART clinics, and longer travel from home to health centers were factors influencing patient satisfaction with antiretroviral treatments. This suggests that further improvement of accessibility is likely needed to increase patient satisfaction.

## Background

Having claimed the lives of more than 20 million peoples in the last three decades, the Human Immunodeficiency Virus (HIV) is the leading cause of morbidity and mortality worldwide [1–3]. Currently, more than 36 million people the majority of whom are from Sub-Saharan countries are living with HIV/AIDS [1, 4]. East and southern African countries share the highest burden of the disease with an estimated 19 million people, nearly 51.7% of the global burden living with it [1, 3]. Globally, the provisions of HAART have shown remarkable progress by reducing HIV/AIDS-related death and new infection by 45 and 23%, respectively in the last 10 years [5].

Although Ethiopia is one of the most affected highly affected Sub-Saharan countries, the national HIV prevalence dropped from 1.4 in 2005 to 0.9% in 2016 despite the regional variations ranging from 1.2 in Amhara to 4.2% in the Gambella region, Ethiopia [6]. According to a recent report, HIV/AIDS-related deaths and new infections decreased by 57 and 5%, respectively from 2010. However, tremendous efforts are still necessary to further control HIV/AIDS spread in the community and to realize the envisioned target of 90–90-90 by the end of 2020, given that only 79% of knew their HIV status of whom 65% of them are on ART treatment. Moreover, Ethiopia launched a five-year national HIV/AIDS strategic plan (2015–2020) based on the investment framework strategy to control HIV/AIDS [2, 7]. Although antiretroviral therapy showed a marked effect on the reduction of death, opportunistic infections, and transmission, only 21.7 million (59%) of the estimated patients were receiving highly active antiretroviral therapy (HAART) by the end of 2017 [1, 4, 8]. In addition, safe and effective ART contributed to improved quality of life, increased survival, decreased HIV-related deaths, prevented the emergence of drug resistance, and decreased social crisis from a number of orphanages [9]. Moreover, the introduction of antiretroviral therapy transformed HIV/AIDS from its nature of high virulence to a manageable health condition.

Patient satisfaction is an indispensable health service outcome measurement which defined as the perceived fulfillment of patient needs and desires through the delivery of healthcare services [10]. It reflects providers’ ability to successfully deliver care that meets patient expectations and needs. Satisfaction is a participatory measurement that helps in a better understanding of the patient-physician relationship and quality of service to the extent of patient needs fulfillment, more importantly for patients on chronic diseases such as HIV/AIDS follow up. Besides, patient satisfaction is an integral part of the health system management strategies to assess the performance of the health system for quality assurance and accreditations [11]. Patient satisfaction is evaluated from the dimensions of the health facility environment, patient-physician relationships, and service availability [12–15]. Moreover, satisfaction among HIV/AIDS patients helps in good adherence and favorable outcome of HAART such as backstage distributions, survival, and low incidence of opportunistic infections (OI) [16]. Socio-demographic characteristics (residence, educational level), clinical conditions such as presence of OI, and hygiene and sanitation of health facilities were factors that influence patient satisfaction [11, 16–19]. The Federal Democratic Republic Ministry of Health of Ethiopia launched a new initiative which required service providers to be Compassionate, Respectful and Caring (CRC) in order to improve patient-physician relationship and increase patient satisfaction from health care services [20]. In addition, optimized and sustained quality of care and treatment are the objectives of the five-year HIV/AIDS strategic plan Ethiopia, which aimed to improve patient satisfaction with HAART services at the health facilities. Most patient satisfaction assessments done among health facilities of Ethiopia were from the perspectives of general services. To date, there have been no patient satisfaction assessments in the health centers which are the components of primary health care units in the Ethiopia health system.

Therefore, this study aimed to assess the level of patient satisfaction with antiretroviral therapy services and it’s determinants at Gondar town health facilities. This study could evaluate the progress of quality service provision to HIV/AIDS patients. In addition, this study could help to increase the decentralization of services by minimizing perceived gaps between patient service needs and the delivery. Furthermore, it might assist in the planning and development of patient satisfaction related to quality improvement projects in ART services at the health center level.

## Methods

### Study design, period and setting

An institution-based cross-sectional study was conducted on four health centers in Gondar town from November 1 to 30, 2018. The town is located in northwest Ethiopia 740 km from the capital Addis Ababa. It has eight public health centers and one comprehensive specialized hospital. Four of the health centers provide antiretroviral therapy services to more than 3000 HIV/AIDS patients in the town and nearby districts. Each health center had a separate ART clinic with its own pharmacy and laboratory units specifically designed to serve only HIV infected patients enrolled for antiretroviral treatment. In addition, each unit has adherence and psychosocial support staff assigned to improve adherence and disclosure.

### Source and eligible population

All adult HIV/AIDS patients who had antiretroviral treatment follow-ups at Gondar health centers were the source population. Similarly, all adult HIV positive people who were on antiretroviral treatment follow-ups for at least 6 months and available during data collection were eligible to take part in the study. Patients who were severely ill and had mental disorders were excluded.

### Sample size determination and sampling procedure

The sample size was determined by using the single population proportion with the assumptions of 80% power, 4% margin of error and 41.5% proportion of satisfaction among HIV/AIDS patients from a study done in Nigeria [21], by considering a 10% non-response rate. The final sample was 663 adult HIV/AIDS patients who were on ART at least for 6 months. Following the opening of HIV/AIDS care and treatment, Gondar, Azezo, Maraki, and Teda health centers enrolled 2160, 768, 381 and 295 ART clients, respectively. The sample was proportionally assigned to each health center based on the number of patients on follow-ups. Accordingly, 398, 141, 70 and 54 ART clinic visitors were systematically selected from Gondar, Azezo, Maraki and Teda health centers, respectively.

### Data collection procedures

Data were collected through a face-to-face interviewer-administered structured questionnaire and document reviews. The questionnaire was initially developed in English after reviewing relevant literature and translated into the local language (Amharic) and back into English to ensure consistency. The questionnaire contained socio-demographic, clinical characteristics and satisfaction measurement domains. The structured questionnaire was pre-tested on 5% of the total sample in a similar setup out of the study area to assure the validity and consistency of the tool. Training was given to four data collectors and two supervisors on how to interview patients using a structured questionnaire and to check the completeness of the questionnaire during interviews. Data about the station and some socio-demographic characteristics such as age and distance travel collected through interview, whereas clinical characteristics like ART regimen they took, recent viral load and opportunistic infections were harvested from patient medical charts and databases. A data collector checked the completeness of each questionnaire at the end of each interview.

### Measurements and operational definitions

In this work, we used 20 questions of the five-point Likert scale, which had domains like the health center physical environment, health care workers’ communication and relationship, accessibility and availability of services to assess patient satisfaction. In the Likert scale rating, 1, 2, 3, 4 and 5 were given for “very dissatisfied”, “dissatisfied”, “neutral”, “satisfied”, and “very satisfied”, respectively. These 20 satisfaction measurement items are scales used to assess the level of satisfaction with antiretroviral therapy services provided in the ART clinics of the town. Since there is no standardized satisfaction measurement tool, we adopt it from previous studies and general services reform measurement perspectives. The Cronbach’s alpha test was used to assess the internal consistency of the satisfaction measurement tool which turned out to be 86%.

Patient satisfaction with antiretroviral therapy services is the response variable, whereas socio-demographic characteristics (age, sex, residence, level of education, religion, occupation, and monthly income), clinical conditions (duration on antiretroviral therapy, recent viral load, ART regimen, adherence status and presence of OI during visits), and physical environment of health center (availability of signs and directions to ART clinic, availability of prescribed ancillary drugs, distance of health facility from living area of the patient and study health center) were the independent variables.

Satisfaction: the degree of concordance between patient expectations of ideal care and their perceptions of real care received.

Satisfied: patient who scored above 60% on the satisfaction measuring items [22].

Dissatisfied: patients who scored 60% and below among the satisfaction measuring items [22].

Opportunistic infection: An infection caused by pathogens like bacteria, viruses, fungi or protozoa that take advantage of low immunity status not normally available.

Good drug adherent: if the average adherence was equal to or greater than (95% or < 3 doses per month) [23].

Fair drug adherent: (85–94% or 4–8 doses missed per month) [23].

Poor drug adherent: (less than 85% or > 9 doses missed per month) [23].

### Data management, processing, and analysis

The completed questionnaire was checked for completeness, consistency and then cleaned and coded by the principal investigator. Data entry was done using EPI data version 3.1 and exported to STATA 14 software for further analysis. Summary statistics like means, medians, and proportions were computed and tables and graphs were used to present them. The five-point Likert scales used to measure patient satisfaction were summarized and ranged between a minimum of 20 and a maximum of 100. Assumptions of normality were checked and the distribution was not normal. We dichotomized the level of satisfaction into two as “satisfied” and “dissatisfied” with a median cutoff point of 60. A binary logistic regression model was fitted to identify factors influencing patient satisfaction with antiretroviral therapy services. Crude and adjusted odds ratios with a 95% CI were computed to assess the strength of associations between independent variables and patient satisfaction. Variables with less than 0.05 *p*-values in multivariable logistic regression analysis were considered as statistically significant associations.

## Result

### Socio-demographic characteristics

A total of 663 adult HIV/AIDS patients were included in the study with the response rate of 100%. The majority (60%) of the participants were from the Gondar health center, followed by 21.3% from Azezo, and the remaining 10.6 and 8.1% from Maraki and Teda health centers, respectively. The mean (SD) age of participants was 37.8 (±9.8) years; more than half (54%) were aged below 37 years, with females accounting for 70.1%. Most (88.1%) of the participants were Orthodox Christians, 93.1% were urban dwellers and only 42.7% were married. Nearly one third (31.4%) were daily laborers (Table 1). Regarding the health center environment, 50.2% of patients saw signs and directions to ART clinics and most (87.5%) of them traveled less than 1 hour to get ART services. Three hundred thirty-three (52%) patients got prescribed ancillary drugs for opportunistic infections medications at pharmacies (Table 1).

**Table 1:** Socio-demographic characteristics of respondents on patient satisfaction and associated factors towards antiretroviral therapy service at Gondar town health center, northwest Ethiopia, 2018 (*n* = 663)

Characteristics	Category	Frequency	Percentage
Age group	<=37	358	54
	38–47	204	30.77
	> = 48	101	15.23
Sex	Male	198	29.9
	Female	465	70.1
Marital status	Single	81	12.2
	Married	283	42.7
	Divorced	205	30.9
	Widowed	94	14.2
Level of education	No formal education	335	50.6
	Primary and secondary school	274	41.3
	College and above	54	8.1
Residence	Urban	617	93.1
	Rural	47	6.9
Religion	Orthodox	584	88.1
	Muslim	61	9.2
	Protestant	11	1.7
	Others	7	1.1
Occupation	Student	21	3.1
	Government employee	92	13.9
	Private worker	132	19.9
	Daily labor	208	31.4
	Merchant	85	12.8
	Housewife	125	18.9
Monthly income (ETB)	No income	24	3.6
	<=1200	322	48.6
	1201–2400	197	29.7
	> = 2401	120	18.1

### Clinical characteristics of patients

The mean (SD) duration in which HIV/AIDS patients received ART was 70.4(±42) months; about 54.45% of the patients were on ART for more than 61 months; half (51.6%) of the patients received TDF/3TC/EFV antiretroviral treatment regimen; most (95.6%) of them had recent viral load measurement of below 1000 copies/ml. About one fourth (25.3%) of the patients had opportunistic infections during the data collection. Four hundred nine (61.7%) of the respondents had good adherence status (Table 2).

**Table 2:** Clinical Characteristics of respondents on patient satisfaction and associated factors towards antiretroviral therapy services at Gondar Town Health Center, Northwest Ethiopia, 2018 (*n* = 663)

Variables	Category	Frequency	Percentage
Duration on ART	≤60 Months	302	45.55
	> 60 Months	361	54.45
Recent viral load	< 1000 copy/ml	634	95.6
	> = 1000 copy/ml	29	4.4
Adherence status	Good	409	61.7
	Fair	158	23.8
	Poor	96	14.5
Opportunistic infections	Yes	168	25.3
	No	495	74.7
ART regimen	AZT/3TC/NEV	120	18.1
	TDF/3TC/EFA	342	51.6
	AZT/3TC/EFA	100	15.1
	TDF/3TC/NEV	101	15.2

AZT/3TC/NEV: Zidovudine/Lamivudine/Neverapin TDF/3TC/EFV: Tenofovir/ Lamivudine/Efavirenz.

AZT/3TC/EFA: Zidovudine/Lamivudine/ Enfavirez TDF/3TC/NEV: Tenofovir/ Lamivudine/ Efavirenz.

### Patient satisfaction with antiretroviral therapy (ART) services

This study revealed that the overall patient satisfaction with antiretroviral service was 75.4% with a 95%CI: 71.9–79). The overall satisfaction specific to each health center ranged from 71.4 to 88.9%. Generally, most of the ‘response was highly (97.1%) satisfied with the convenience of time to get ART services and (91.5%) convenience situations and (90.8%) with easy accessibility of drugs at pharmacies. On the other hand, (40.7%) of the patients showed low satisfaction with the availability and accessibility of latrines and (27.3%) with the cleanness and comfortability of latrines at the health centers. Detail patient satisfaction measurements mentioned in (Table 3).

**Table 3:** Rate of patients satisfaction on ART service by different measuring items in Gondar Town ART Health Centers, North wet Ethiopia,2018 (*n* = 663)

S.N	Variables	V. dissatisfied	dissatisfied	Neutral	satisfied	v. satisfied	Mean (SD) rating
		No (%)	No (%)	No (%)	No (%)	No (%)	
1	Staff treats all patients fairly and equally	96 (14.5)	79 (11.9)	67 (10.1)	375 (56.6)	46 (7.1)	3.29(±1.20)
2	Health professional compassion and support the patient	22 (3.3)	67 (10.1)	61 (9.2)	462 (69.7)	51 (7.7)	3.68(±0.87)
3	ART clinic staffs availability on working hours	130 (19.6)	86 (13)	21 (3.2)	393 (59.3)	33 (5)	3.17(±1.29)
4	Privacy during examination	14 (2.1)	31 (4.7)	96 (14.5)	454 (68.5)	68 (10.3)	3.80(±0.76)
5	Health professional respect the patient and courtesy	39 (5.9)	58 (8.7)	46 (6.9)	454 (68.5)	66 (10)	3.67(±0.97)
6	Health professional obligate and adoration ethics	43 (6.5)	61 (9.2)	50 (7.5)	457 (68.9)	52 (7.8)	3.62(±0.98)
7	Cleanness of health center and rooms	56 (8.4)	126 (19)	73 (11)	377 (56.9)	31 (4.7)	3.30(±1.09)
8	Comfort ability of seating chair	68 (10.3)	153 (23.1)	66 (10)	353 (53.2)	23 (3.5)	3.16(±1.13)
9	Comfort ability of waiting and examination room	63 (9.5)	133 (20.1)	112 (16.9)	351 (52.9)	4 (0.6)	3.15(±1.05)
10	Availability and accessibility of latrine	116 (17.5)	202 (30.5)	75 (11.3)	266 (40.1)	4 (0.6)	2.75(±1.17)
11	Cleanness and comfort ability of latrine	201 (30.3)	187 (28.2)	94 (14.2)	177 (26.7)	4 (0.6)	2.39(±1.19)
12	Drug available during visit	29 (4.4)	78 (11.8)	13 (2)	448 (67.6)	95 (14.3)	3.75(±0.98)
13	Laboratory tests available during the visit	71 (10.7)	107 (16.1)	23 (3.5)	380 (57.3)	82 (12.4)	3.44(±1.20)
14	Sufficiency of service giving rooms	39 (5.9)	92 (13.9)	47 (7.1)	438 (66.1)	47 (7.1)	3.54(±(1.01)
15	Availability of good diagnosis	61 (9.2)	44 (6.6)	28 (4.2)	449 (67.7)	81 (12.2)	3.67(±1.07)
16	Easily obtaining drugs at pharmacies during visiting	23 (3.5)	21 (3.2)	17 (2.6)	502 (75.7)	100 (15.1)	3.95(±0.78)
17	Convenient situations to get drugs from pharmacy	6 (0.9)	39 (5.9)	11 (1.7)	509 (76.8)	98 (14.8)	3.98(±0.69)
18	Convince and safety of ART clinic for visiting	58 (8.7)	83 (12.5)	23 (3.5)	445 (67.1)	54 (8.1)	3.53(±1.09)
19	Distance of Health center from leaving area	32 (4.8)	25 (3.8)	48 (7.2)	491 (74.1)	67 (10.1)	3.80(±0.85)
20	Convenience of time to get ART services	4 (0.6)	5 (0.8)	10 (1.5)	587 (88.5)	57 (8.6)	4.03 (0.42)
Overall level of satisfaction by health centers (satisfaction > 60)				Satisfied n (%)		Mean (SD) rating	
1	Gondar health center			284 (71.4)		3.43(±0.56)	
2	Maraki health center			50 (71.4)		3.53(±0.77)	
3	Azezo health center			118 (83.7)		5.59(±0.49)	
4	Teda health center			48 (88.9)		3.59(±0.48)	

### Factors affecting patient satisfaction on antiretroviral therapy service

For patients who are aged between 38 to 47 years or above 48 years, the odds of satisfaction were 5.9 (AOR = 5.90;95% CI:3.38–10.31) and 2.66 (AOR = 2.66;95% CI:1.38–5.12) times higher, respectively, compared to those aged below 37 years. For patients who traveled more than 1 h to get services, the odds of satisfaction was decreased by 44% compared to those who traveled less (AOR = 0.56; 95% CI: 0.32, 0.97). For those who did not notice signs and directions to antiretroviral therapy clinics, the odds of satisfaction dropped by 47% compared to those who saw signs and directions to service units (AOR = 0.53;95% CI:0.35,0.82). Compared to patients who had to follow-ups at the Gondar health center, the odds of satisfaction at Azezo and Teda health centers were 2.62 **(**AOR = 2.68; 95%CI: 1.47 2.48) and 4.44 (AOR = 4.4; 95%CI: 1.73, 11.30**)** times higher (Table 4).

**Table 4:** Bi-variable and multivariate analysis of predictors to assess factors affecting patient satisfaction on antiretroviral therapy services at Gondar town health centers, northwest Ethiopia,2018(*n* = 663)

Variables	Patient satisfied		Crude OR(95%CI)	Adjusted OR(95%CI)
	Yes	No		
Age category				
< =37	228	130	1	1
38–47	186	18	5.89 (3.46, 10.00)	5.90 (3.38, 10.31)*
> =48	86	15	3.26 (1.81, 5.89)	2.66 (1.38, 5.12)*
Sex				
Male	156	42	1	1
Female	344	121	0.76 (0.51–1.14)	0.76 (0.51, 1.14)
Marital status				
Single	54	27	1	1
Married	209	74	1.41 (0.82–2.40)	1.04 (0.57, 1.88)
Divorced	159	46	1.73 (0.98–3.04)	1.34 (0.71, 2.53)
Widowed	78	16	2.43 (1.21–4.90)	1.52 (0.66, 3.48)
Educational status				
No formal education	259	76	1	1
Primary and secondary school	199	75	0.77 (0.53–1.12)	0.76 (0.51, 1.15)
Collage and above	42	12	1.02 (0.51–2.04)	0.88 (0.41, 1.91)
Duration on ART				
≤ 60 months	220	82	1	1
≥ 61 months	280	81	1.28 (0.90–1.83)	1.03 (0.69–1.53)
Adherence status				
Good	313	96	1	1
Fair	121	37	1.00(0.65–1.54)	0.92 (0.57,1.48)
Poor	66	30	0.67 (0.41–1.10)	0.84 (0.48,1.46)
Availability of signs and directions to the ART clinic				
Yes	258	75	1	1
No	242	88	0.79 (0.56–1.13)	0.53 (0.35,0.82)*
Distance travel from home to health centers				
≤ 1 h	446	134	1	1
> 1 h	54	29	0.55 (0.34–0.91)	0.56 (0.32,0.97)*
Study health centers				
Gondar Health Center	284	114	1	1
Maraki Health Center	50	20	1.04(0.57–1.76)	1.27 (0.67,2.43)
Azezo Health Center	118	23	2.05 (1.25–3.380	2.68 (1.47,4.66)*
Teda Health Center	48	6	3.21(1.33–7.71)	4.44 (1.73,11.30)*

* Statistical significant at a *p*-value of 0.05, *ART* Antiretroviral Treatment

## Discussion

The overall patient satisfaction, which included significant variations ranging from 71.4 to 88.9% among health centers, was 75.4% in Gondar. Age of respondents, absence of signs and directions to ART clinic, and longer travel from home to health centers were factors influencing patient satisfaction with ART services. Significant variations among health centers might be explained by differences in the setups, health professional experiences, and materials availability, and accessibility in the health centers. The level of satisfaction in this study was consistent with that of a study at a referral hospital in Ethiopia (75.2%) [14] and a public hospital in Nigeria (71.4%) [21]. Patients satisfaction measurement had important implications for services providers and planners to identify gaps and improvement of service quality of the health care system.

However, this finding was lower than results of health centers in Tigray region, Ethiopia (89.6%) [22], Addis Ababa public hospitals (85.5%) [24], Sidama hospital, southern Ethiopia (85.5%) [15], and Bamenda-Cameroon 91.2% [19]. The reason could be the differences in the study setting, socio-demographic characteristics, and service units. In addition studies in Addis Ababa and Sidama hospitals, patients satisfaction measurement was focused on laboratory services. Furthermore, hospitals had better diagnostic and medical personnel for better health care service provision which fulfills patient expectations compared to health centers.

On the other hand, this study finding was also higher compared to the result at the University of Gondar hospital which assessed satisfaction with ART laboratory services (54.7%) and Tanzania (65.2%) [12, 25]. The possible explanations might be differences in study settings, quality of service and units assessed for satisfaction such as laboratories. A study done in Tanzania included private health facilities which might have affected the level of satisfaction.

As shown in Table 3, among domains used to assess satisfaction, the convenience of time to get services and easy accessibility of pharmacies attracted high satisfaction scores, In contrast, domains related to health center physical environment such as availability, accessibility and cleanness, and comfort of latrines received lower ratings. This could be due to the fact that poor environmental hygiene in health facilities is associated with increased occurrence of nosocomial infections which might affect the safety of immunocompromised patients. This finding showed that higher satisfaction (97.1%) was reported on the convenience of time to get services, the convenience of situations to get drugs from pharmacies (91.5%) and distance of health center from living area 84.2%. These findings were consistent with those of other studies conducted at government hospitals of eastern Ethiopia [26].

This study showed that patients who were older than 38 years were associated with increased satisfaction with antiretroviral treatment services compared to younger ones. This was incongruent with results in Ethiopia and Nigeria. HIV/AIDS is a stigmatizing disease that affects social interactions; thus, older patients are psychologically stable and more satisfied with services provided at health centers [14, 17, 27]..

For patients who traveled more than 1 h from home to health centers, satisfaction decreased by 44% compared to those patients who traveled less than 1 h to get to antiretroviral therapy service health centers. This result was consistent with those of studies in the Tigray region and the public health facility of the Sidama zone of Ethiopia [16, 22]. The possible explanations might be the fact that long-distance travel is often associated with increased financial expenditure and absence from work and school.

Patients who did not observe signs and directions to antiretroviral therapy units were associated with lower rates of patient satisfaction compared to those who observed signs and directions. This finding was consistent with that of a previous study in Ethiopia. The possible reason for the decreased satisfaction could be that directions to ART clinics help patients to get where ART clinics are located. That is because patients fear the stigma and discrimination to ask other people to show them where the ART clinic is found [13].

This study has implications for patients, health care workers, public health experts and health system administrators for the improvement of health services quality and increasing patient retention on the HAART. In addition, factors identified like distance travel and sign and direction of health facilities suggest the need to scale up of health service accessibility and availability through further decentralization of services [28].

Although this study used a large sample size and multi-center approach, it has limitations related to the lack of the qualitative component which is important for assessing patient insight and beliefs. Besides the satisfaction measurement tool which is not standardized and might introduce misclassifications and bias. Moreover, this study result may not show the satisfaction of patients who had follow-ups in hospitals. Finally, the dichotomization of Likert scale measurements might introduce misclassification and disruption of the data nature.

## Conclusion

The overall patient satisfaction with antiretroviral therapy service was lower than the national target of 85% with the marked difference among health centers. Older age, absence of signs and directions to ART clinics, and longer travel from home to health centers were factors influencing patient satisfaction with antiretroviral treatments. The findings strongly suggest that programmatic/ health policy decisions related to increased access to ART in the region. This suggests that further improvement of accessibility is mandatory for increasing patient satisfaction.

## Data Availability

The datasets used during the current study are available from the corresponding author on a reasonable request.

## Abbreviations

AIDS: Acquired Immune deficiency Syndrome
AOR: Adjusted Odds Ratio
ART: Antiretroviral Therapy
CI: Confidence Interval
CRC: Compassionate Respectful and Caring
FMOH: Federal Ministry of Health
HAART: Highly Active Antiretroviral Therapy
HC: Health Center
HIV: Human Immunodeficiency Virus
OI: Opportunistic Infection
SD: Standard deviation
